# The effect of Q.Clear reconstruction on quantification and spatial resolution of 18F-FDG PET in simultaneous PET/MR

**DOI:** 10.1186/s40658-021-00428-w

**Published:** 2022-01-10

**Authors:** Defeng Tian, Hongwei Yang, Yan Li, Bixiao Cui, Jie Lu

**Affiliations:** 1 Department of Radiology and Nuclear Medicine, Xuanwu Hospital, Capital Medical University, 45# Changchun Street, Xicheng District, Beijing, China; 2 Beijing Key Laboratory of Magnetic Resonance Imaging and Brain Informatics, Beijing, China

**Keywords:** PET/MR, Q.Clear, Penalization factor *β*, OSEM

## Abstract

**Background:**

Q.Clear is a block sequential regularized expectation maximization penalized-likelihood reconstruction algorithm for Positron Emission Tomography (PET). It has shown high potential in improving image reconstruction quality and quantification accuracy in PET/CT system. However, the evaluation of Q.Clear in PET/MR system, especially for clinical applications, is still rare. This study aimed to evaluate the impact of Q.Clear on the ^18^F-fluorodeoxyglucose (FDG) PET/MR system and to determine the optimal penalization factor *β* for clinical use.

**Methods:**

A PET National Electrical Manufacturers Association/ International Electrotechnical Commission (NEMA/IEC) phantom was scanned on GE SIGNA PET/MR, based on NEMA NU 2-2012 standard. Metrics including contrast recovery (CR), background variability (BV), signal-to-noise ratio (SNR) and spatial resolution were evaluated for phantom data. For clinical data, lesion SNR, signal to background ratio (SBR), noise level and visual scores were evaluated. PET images reconstructed from OSEM + TOF and Q.Clear were visually compared and statistically analyzed, where OSEM + TOF adopted point spread function as default procedure, and Q.Clear used different *β* values of 100, 200, 300, 400, 500, 800, 1100 and 1400.

**Results:**

For phantom data, as *β* value increased, CR and BV of all sizes of spheres decreased in general; images reconstructed from Q.Clear reached the peak SNR with *β* value of 400 and generally had better resolution than those from OSEM + TOF. For clinical data, compared with OSEM + TOF, Q.Clear with *β* value of 400 achieved 138% increment in median SNR (from 58.8 to 166.0), 59% increment in median SBR (from 4.2 to 6.8) and 38% decrement in median noise level (from 0.14 to 0.09). Based on visual assessment from two physicians, Q.Clear with *β* values ranging from 200 to 400 consistently achieved higher scores than OSEM + TOF, where *β* value of 400 was considered optimal.

**Conclusions:**

The present study indicated that, on ^18^F-FDG PET/MR, Q.Clear reconstruction improved the image quality compared to OSEM + TOF. *β* value of 400 was optimal for Q.Clear reconstruction.

## Introduction

Positron Emission Tomography (PET) with ^18^F-fluorodeoxyglucose (FDG) is a powerful imaging technique in oncology studies. PET image quality is affected by both hardware specifications and reconstruction algorithms. In the past, filtered back-projection (FBP) was used to reconstruct PET images. Lately, statistical iteration methods including maximum likelihood expectation maximization (MLEM) have been developed. Iterative reconstruction techniques generally performed better than standard analytical methods because they could achieve higher signal-to-noise (SNR). Currently, the most widely used PET reconstruction algorithm for clinical data is the Ordered Subsets Expectation Maximization (OSEM). Nevertheless, OSEM has an inherent drawback; it cannot achieve full convergence due to increased noise in the image with the increase in iteration times. As a result, OSEM algorithm is usually stopped after two to four iterations to avoid bringing excessive noise in images, which results in under-convergence image and brings bias in lesion quantification.

To address the under-convergence effects and to improve quantification accuracy, a Bayesian penalized likelihood reconstruction algorithm named Q.Clear (GE Healthcare) has recently been introduced. As a block sequence regularization expectation–maximization (BSREM) penalty likelihood reconstruction algorithm, Q.Clear can achieve full convergence and provide a more accurate quantitation and higher SNR than OSEM. Several studies comparing Q. Clear and OSEM have been reported recently. However, most of these studies were conducted on PET/CT but not PET/MR, and the very few studies conducted on PET/MR used phantom data only. When it comes to integrated PET/MR, the PET imaging environment becomes more complex than that in PET/CT. In PET/MR, PET imaging could be affected by physical environmental factors, including magnetic field, radiofrequency field and gradient fields. Another difference is the attenuation correction (AC) method, where PET/MR needs segmented MR images to generate pseudo-CT images, while PET/CT can derive AC map from CT data directly. Recently, two studies have been published on Q.Clear in PET/MR and both concluded that Q.Clear achieved better image quality than OSEM [1, 2]. However, neither studies simultaneously performed clinical and phantom measurements. While the optimal penalization factor (*β*) for Q.Clear has been investigated on PET/CT [3–6], a study on PET/MR with both phantom and clinical data evaluation was still necessary. In this study, we aimed to conduct a comprehensive evaluation of Q.Clear for the ^18^F-FDG PET/MR images reconstruction, using various metrics including quantification accuracy, detectability and image quality.

## Materials and methods

### Phantom

#### Phantom preparation

A National Electrical Manufacturers Association/International Electrotechnical Commission (NEMA/IEC) body phantom was used to evaluate image quality in the current study [7]. This phantom contains six spheres with different diameters of 10 mm, 13 mm, 17 mm, 22 mm, 28 mm and 37 mm, to simulate lesions of different sizes. A lung insert filled with low-density styrofoam pellets and pure water was positioned in the center of the phantom to simulate human lung tissue. The two largest spheres (28 mm and 37 mm) simulating cold lesions were filled with non-radioactive water, while the four smallest spheres (10 mm, 13 mm, 17 mm and 22 mm) simulating hot lesions were filled with 18F-FDG at an activity concentration ratio four times to the background [7]. To get homogenous activity concentration and the fewest bubbles, the phantom was left fully standing after filling. Measurement started when the ^18^F-FDG activity concentration of the background reached 5.2 kBq/ml.

#### Phantom TOF PET/MR imaging protocol and image reconstruction

All scans were performed on a GE SIGNA PET/MR scanner (MP26). The PET/MR system features a simultaneous time of flight (TOF) PET imaging integrated with whole-body 3.0-T MRI scanner. The PET detectors provide a 25-cm axial field of view (FOV) and a 60-cm trans-axial FOV. The TOF timing resolution is 386 psec [8]. Three phantom scans were performed to assess the variations. The acquisition time of the PET image was 12 min. A matrix size of 192 × 192 was used, resulting in a voxel size of 2.08 × 2.08 × 2.78 mm^3^. Attenuation and scatter corrections were performed.

All images were reconstructed on a GE AW4.7 workstation. OSEM used the following parameters: 3 iterations, 28 subsets; point spread function (PSF) modeling; Gaussian low-pass filter of 4.0 mm FWHM; with TOF (OSEM + TOF). Q.Clear used TOF and the following *β* values: 100, 200, 300, 400, 500, 800, 1100 and 1400.

#### Phantom PET image analyses

A circular region of interest (ROI) was placed on each sphere. Ten circular ROIs of 100 mm^2^ were drawn on the slices at the distance of ± 1 cm and ± 2 cm to the phantom center. Contrast recovery (CR) and background variability (BV) of both hot and cold spheres and SNR of hot spheres were analyzed according to NEMA NU 2-2012 standard [7], based on the following formula (1–5):

1 $${\text{CR}}_{{{\text{H}},j}} = \frac{{\left( {\frac{{C_{{{\text{H}},j}} }}{{C_{{\text{B}}} }}} \right) - 1}}{{\left( {\frac{{a_{{\text{H}}} }}{{a_{{\text{B}}} }}} \right) - 1}} \times 100\%$$

2 $${\text{CR}}_{{{\text{C}},j}} = \left( {1 - \frac{{C_{{{\text{C}},j}} }}{{C_{{\text{B}}} }}} \right) \times 100\%$$

3 $${\text{SD}}_{j} = \sqrt {\mathop \sum \limits_{k = 1}^{K} \frac{{\left( {C_{{{\text{B}},k}} - C_{{\text{B}}} } \right)^{2} }}{{\left( {K - 1} \right)}}} ,\quad K = 60$$

4 $${\text{BV}}_{j} = \frac{{{\text{SD}}_{j} }}{{C_{{\text{B}}} }} \times 100\%$$

5 $${\text{SNR}}_{{{\text{H}},j}} = \frac{{C_{{{\text{H}},j}} - C_{{\text{B}}} }}{{{\text{SD}}_{j} }} \times 100\%$$

where $C_{{{\text{H}},j}}$ is the average counts in the ROI for hot spheres; $C_{{\text{B}}}$ is the average of 60 background ROI counts for spheres; $a_{{\text{H}}}$ is the activity concentration in the hot spheres and $a_{{\text{B}}}$ is the activity concentration in the background; ${\text{CR}}_{{{\text{C}},j}}$ is the average counts in the ROI for cold spheres;${\text{SD}}_{j}$ is the standard deviation of the background ROI counts for spheres; *K* equals the 60 background ROI counts.

#### Spatial resolution

The full width at half maximum (FWHM) was assessed in different reconstruction results [7]. It was measured by three-point sources of the capillary tube in the air, with ^18^F-FDG radioactivity concentration of 200 MBq/ml in the end of glass capillary tubes. Data were collected in the central plane of FOV, with at least 500,000 counts for each scan.

### Clinical evaluation

#### Case selection

Informed consent was not obtained due to the retrospective nature of this study. Data were anonymized before analysis. Patients were asked to fast for at least four hours before the scan, and their blood glucose level was under 8.0 mmol/l. All PET/MR examinations were performed from skull base to knees or feet (whole body). In this study, 10 consecutive patients with torso cancer were retrospectively selected between August 2020 and March 2021, which included 7 women and 3 men, with a median age of 63 year. All patients were scanned for at least 5–6 bed positions with 6 min per bed position (min/bp). The administrated ^18^F-FDG was 3.7 MBq/kg, and the uptake time between administration and imaging was 30–36 min.

#### Clinical TOF PET/MR imaging protocol and image reconstruction

Reconstruction method was the same as in phantom study.

#### PET image analyses

Noise level, lesion SNR and signal to background ratio (SBR) were calculated. OSEM + TOF images were used as baseline. Lesions in the volumes of interest VOIs were delineated with a 41% threshold of the maximum voxel value. Noise level was calculated from standard deviation (SD) divided by the mean standardized uptake values (SUVmean) of a large spherical reference VOI (3.0 cm) placed in the normal liver. The lesion SNR was calculated from lesion’s max standardized uptake values (SUVmax) divided by noise level. SBR was calculated from lesion’s SUVmax divided by SUVmean of the liver reference VOI background.

Two nuclear medicine physicians with more than 20 years of experience in PET diagnosis independently evaluated PET images reconstructed from different algorithms in a random order. The physicians were not aware of the reconstruction parameters, and retrospective changes to assessments were not allowed. Lesion detectability and overall image quality were visually assessed with 5-point Likert-like scale [(1) non-diagnostic: inability to discern lesions from background; (2) poor: only subtle distinction of lesions from background; (3) moderate: ability to discern lesions with significant noise; (4) good: ability to discern lesions with low noise; (5) excellent: ability to discern lesions without noise] in accordance with a previously published study [9].

### Statistical analyses

Statistical analyses were conducted on SPSS Statistics 22.0 (IBM Co., New York, USA). Normally distributed data were displayed as mean ± SD, and skewed data were displayed as medians (interquartile ranges—IQRs). A nonparametric test, Friedman test, was performed to identify differences between OSEM + TOF and Q.Clear with specific *β* value. If significant differences (*P* < 0.05) were observed in Friedman test, Bonferroni adjustment was performed for pairwise post hoc comparison (between OSEM + TOF and Q.Clear with specific *β* value). Bonferroni corrected *P* value lower than 0.05 was considered statistically significant.

## Results

### Phantom data

#### *CR, BV and SNR of OSEM* + *TOF and Q.Clear*

**Fig. 1:**
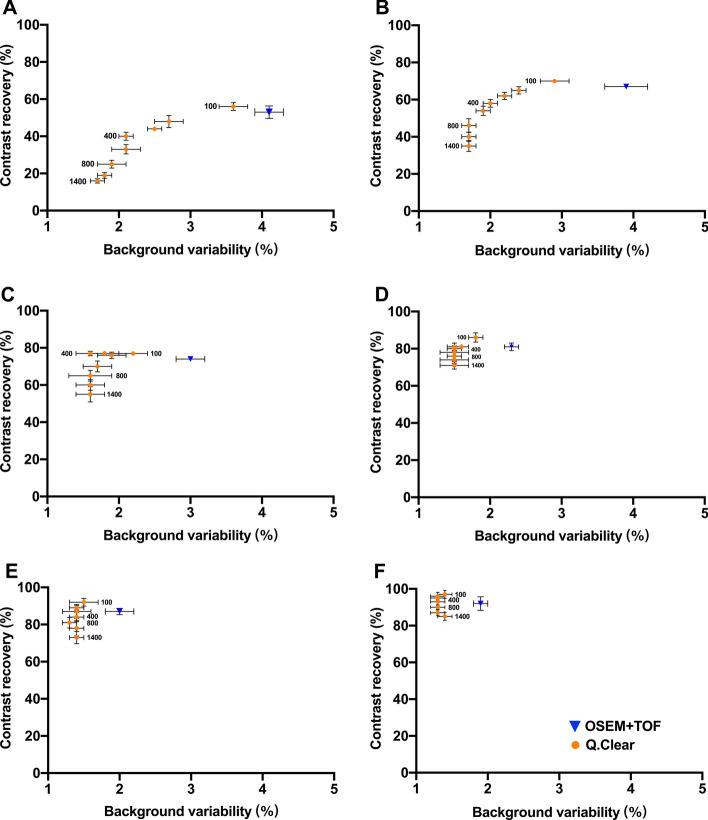
Mean CR and BV for all spheres with diameters of 10 mm (**A**), 13 mm (**B**), 17 mm (**C**), 22 mm (**D**), 28 mm (**E**), 37 mm (**F**). These were shown for OSEM + TOF (3 iterations, 28 subsets, 4.0-mm filter), Q.Clear (*β* = 100, 200, 300, 400, 500, 800, 1100 and 1400, as labeled on the points). Values are presented as means with SDs

**Fig. 2:**
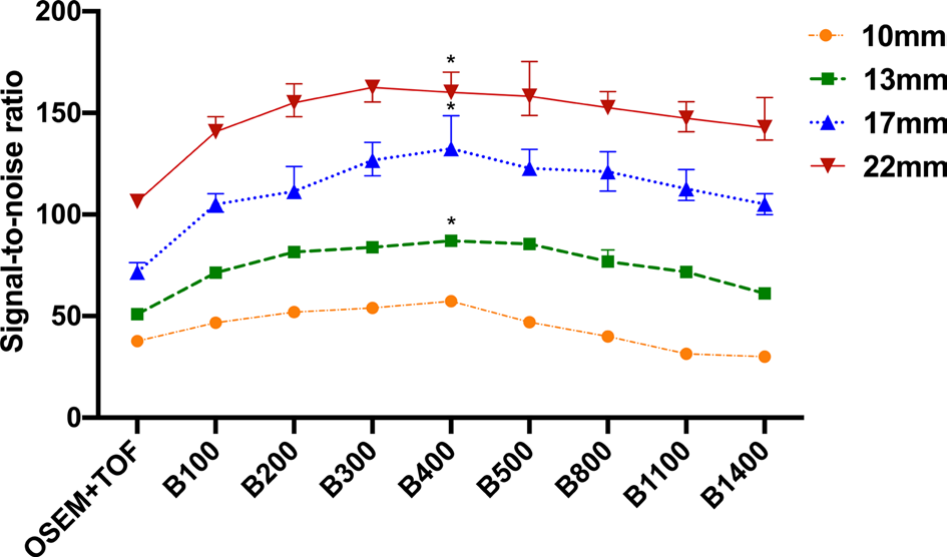
SNR for all hot spheres with diameters of 10 mm, 13 mm, 17 mm and 22 mm. These were shown for OSEM + TOF (3 iterations, 28 subsets, 4.0-mm filter), Q.Clear (*β* = 100, 200, 300, 400, 500, 800, 1100 and 1400). Values are presented as medians (IQRs). * represents a statistical difference compared to OSEM + TOF (Bonferroni corrected *P* < 0.05)

The results for the phantom study are summarized in Fig. 1. As the *β* value increased, CR and BV of all sizes of spheres generally decreased. Generally, Q.Clear always yielded a lower BV than OSEM + TOF. Figure 2 shows SNR for all hot spheres, where SNR_10mm_, SNR_13mm_, SNR_17mm_ and SNR_22mm_ peaked at *β* of 400 and subsequently decreased as *β* increased. SNR values of Q.clear with *β* value of 400 were consistently higher than OSEM + TOF for 13 mm, 17 mm and 22 mm spheres (Bonferroni corrected *P* < 0.05).

#### The spatial resolution

Table 1 summarizes the reconstructed radial, tangential and axial (*X*, *Y*, *Z*) resolutions (FWHM) for each radius (1, 10 and 20 cm) of capillary tube locations embedded in the air for all reconstruction methods. Q.Clear reconstruction with different center radius generally achieved better resolution than OSEM + TOF.

**Table 1:** FWHM for reconstructed spatial resolution

		OSEM + TOF	B100	B200	B300	B400	B500	B800	B1100	B1400
Resolution direction	Radial offset									
Radial	1 cm	4.39	1.69	1.72	1.76	1.80	1.84	2.00	2.18	2.36
	10 cm	4.39	1.78	1.84	1.91	1.98	2.06	2.30	2.50	2.65
	20 cm	5.06	2.99	3.08	3.17	3.33	3.39	3.59	3.74	3.86
Tangential	1 cm	4.35	1.57	1.61	1.65	1.70	1.74	1.91	2.10	2.26
	10 cm	4.35	2.19	2.24	2.28	2.33	2.37	2.48	2.58	2.67
	20 cm	4.83	2.86	3.03	3.14	3.22	3.27	3.40	3.51	3.61
Axial	1 cm	3.88	3.91	3.93	3.95	3.96	3.98	4.04	4.13	4.23
	10 cm	3.88	3.34	3.38	3.42	3.46	3.49	3.60	3.70	3.82
	20 cm	4.82	4.81	4.85	4.89	4.91	4.94	5.00	5.06	5.12

FWHM for the capillary tubes at the direction of radial, tangential, axial at the off-center 1 cm,10 cm and 20 cm. Reconstruction algorithms were presented for OSEM + TOF (3 iterations, 28 subsets, 4.0-mm filter), Q.Clear (*β* = 100, 200, 300, 400, 800, 1100 and 1400)

### Clinical data

#### *SNR, SBR and noise level of OSEM* + *TOF and Q.Clear*

**Fig. 3:**
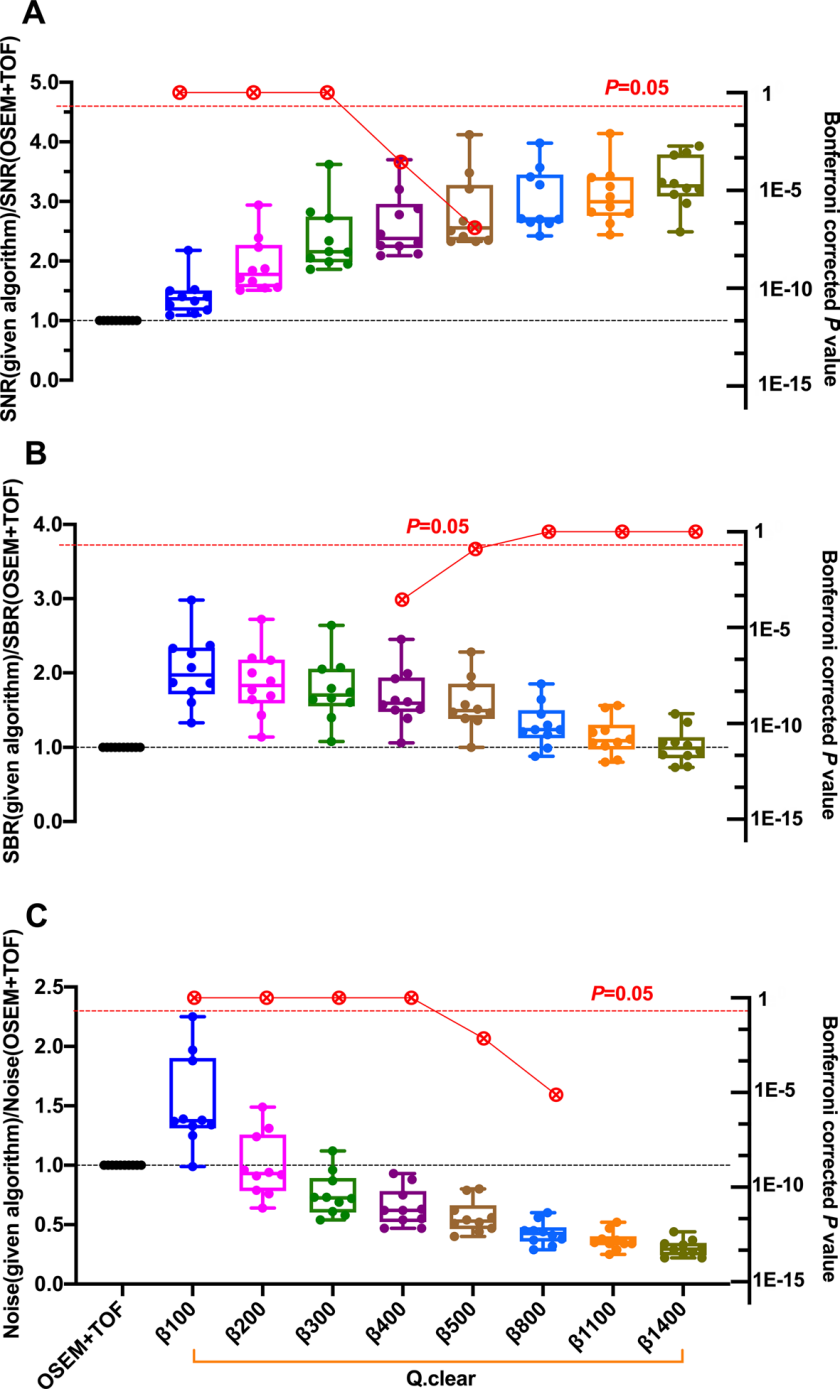
Box plots of SNR (**A**), SBR (**B**) and noise level (**C**) values calculated among the PET images of torso oncologic patients with different reconstruction algorithms. These were shown for Q.Clear (*β* = 100, 200, 300, 400, 500, 800, 1100 and 1400), normalized to OSEM + TOF (3 iterations, 28 subsets, 4.0-mm filter) (left *Y*-axis). The lines, upper and lower halves of the box represent the median, upper and lower quartiles, respectively. Bonferroni corrected *P* values between OSEM + TOF and a given algorithm (Q.Clear with specific *β* value) were also presented in red circles (right *Y*-axis).

Figure 3A–C shows the SNR, SBR and noise level values of Q.Clear with variable *β* values of 100–1400 and normalized to OSEM + TOF reconstruction SNR, SBR and noise level values (left *Y*-axis). Bonferroni corrected *P* values between OSEM + TOF and Q.Clear with a specific *β* value are also presented in Fig. 3A–C (red circles, right *Y*-axis). The lowest noise level was achieved with the highest *β* value, resulting in highest SNR and in turn the lowest SBR. Compared to OSEM + TOF, Q.Clear with *β* value of 400 produced a median of SNR that was increased by 138% (from 58.8 to 166.0), a median of SBR that was increased by 59% (from 4.2 to 6.8) (Bonferroni corrected *P* < 0.05 for both), a median of noise level that was decreased by 38% (from 0.14 to 0.09).

#### Visual assessment scores

**Fig. 4:**
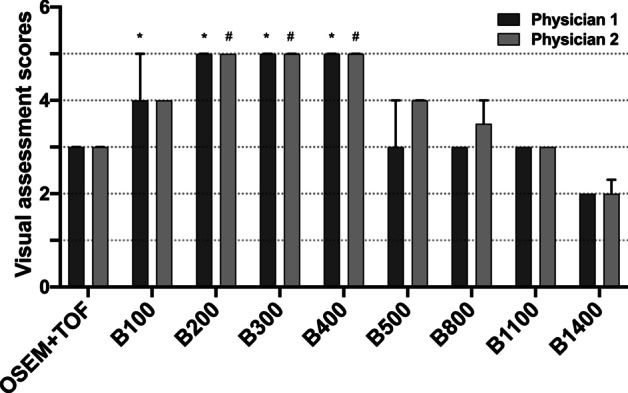
The differences between the visual assessment scores among the PET images of torso oncologic patients with different reconstruction algorithms. These were shown for OSEM + TOF (3 iterations, 28 subsets, 4.0-mm filter), Q.Clear (*β* = 100, 200, 300, 400, 500, 800, 1100 and 1400). Values are presented as medians (IQRs). * and ^#^ represent statistical difference compared to OSEM + TOF for physician 1 and physician 2, respectively (Bonferroni corrected *P* < 0.05)

The results of visual assessment for image quality are shown in Fig. 4. *β* value of 200–400 resulted in excellent image (mean score: 5 out of 5) and OSEM + TOF reconstructions result in moderate image (mean score: 3 out of 5). The summary of both physicians’ evaluation indicated that Q.Clear with *β* value of 200–400 performed better than OSEM + TOF (Bonferroni corrected *P* < 0.05).

## Discussion

We investigated the performance of Q.Clear and OSEM + TOF for the reconstruction of both phantom and clinical PET data acquired on 18F-FDG integrated PET/MR system. For phantom data, our results indicate that PET images reconstructed with Q.Clear algorithms with *β* of 400 result in lower CR and BV, higher SNR values and better spatial resolution than OSEM + TOF. For clinical data, Q.Clear with *β* of 400 achieved a significant improvement in SNR, SBR and noise level compared to OSEM + TOF. The maximal visual assessment scores on clinical image were achieved at *β* ranging from 200 to 400. Overall, we suggest Q.Clear with *β* of 400 as the optimal PET reconstruction setting in PET/MR.

In general, Q.Clear yielded lower BV than OSEM + TOF. BV gradually decreased with increase in *β* value. In contrast, the highest CR was achieved by Q.Clear with the smallest *β* value (*β* = 100). As *β* increased, CR decreased. A choice for *β* had to be made to balance BV and CR. Ideally, these points on the graph should be in the upper left corner of each graph in Fig. 1. In 13-, 17- and 22-mm spheres, Q.Clear images with *β* values of 400 achieved the highest SNR.

Spatial resolution was affected by the noise levels and reconstruction methods. Q.Clear applied to adjacent pixels, and the degree of edge preservation can be controlled through the use of an optimum penalization factor *β*. In general, Q.Clear reconstruction achieved better spatial resolution than OSEM + TOF throughout the FOV (Table 1), which was in accordance with previous studies [10]. The best spatial resolution was achieved with the smallest *β* (*β* = 100) and would degrade as *β* increased. It was probably due to the negative effect of CR reduction on spatial resolution and the inherent smoothing performance of the Q.Clear algorithm. However, in terms of the evaluation of spatial resolution, *β* value of 400 was still acceptable.

Our clinical data analysis also indicated significantly improved SNR, SBR, noise level and visual assessment scores in Q.Clear (*β* = 400) than in OSEM + TOF, which was in line with previously published studies [11–14]. Compared to OSEM + TOF, Q.Clear can achieve a complete convergence and more accurate lesion quantitation, which will improve the SUVmax, SNR, SBR values of PET/MR in patients with suspected primary and metastatic torso cancers. The phantom study showed similarities to clinical data, where the optimal *β* value for both phantom data and for clinical data was 400. Therefore, we suggest that a *β* value of 400 would be an optimal choice. Our findings were similar to previous studies on PET/MR using ^68^ Ga-prostate-specific membrane antigen (400–550) [1]. Previous studies on PET/CT suggested similar optimal *β* value of 350–400 (^18^F-FDG) [12, 15, 16], 300–550 (^18^F-fluciclovine) [17] and 300 (^18^F-NaF) [14], 350 (^68^ Ga-labeled radiopharmaceuticals) [11], 400–550 (^18^F-fluorocholine) [18]. While a few other studies of PET/CT suggested a higher *β* value of 500–750 [19–24], a study on the detection of sub-centimeter lesions suggested a *β* value of 200 [25]. The variations of optimal *β* ascribed to PET data acquisitions could have been affected by many factors, including scanning range, count statistics, radiation dose, the acquisition time and spatial resolution (the axial FOV) of the PET/MR used. Different scan protocols would lead to variations in evaluation metrics such as CR, BV and SNR, which would finally affect the choice of the optimal *β* value.

However, we have to admit that there were some limitations of this study. Firstly, the minimum diameter of the simulated phantom sphere was 10 mm, which could not pair smaller lesions in patients. Besides, only ten patients with metastatic torso cancers were included for this study. More patients with various diseases would form a more comprehensive evaluation and reduce biases.

## Conclusions

The present study indicates that on ^18^F-FDG PET/MR, Q.Clear reconstruction improves the image quality compared to OSEM + TOF. The *β* value of 400 is optimal for Q.Clear reconstruction.

## Data Availability

The datasets used and/or analyzed during the current study are available from the corresponding author on reasonable request.

## Abbreviations

PET: Positron Emission Tomography
FDG: ^18^F-fluorodeoxyglucose
FBP: Filtered back-projection
MLEM: Maximum likelihood expectation maximization
SNR: Signal-to-noise ratio
OSEM: Ordered subset expectation maximization
BSREM: Block sequence regularization expectation–maximization
AC: Attenuation correction
NEMA/IEC: National Electrical Manufacturers Association/International Electrotechnical Commission
TOF: Time of flight
FOV: Field of view
PSF: Point spread function
ROI: Region of interest
CR: Contrast recovery
BV: Background variability
FWHM: Full width at half maximum
SBR: Signal to background ratio
VOI: Volume of interest
SD: Standard deviation
SUVmean: Mean standardized uptake value
SUVmax: Max standardized uptake value
IQRs: Interquartile ranges
